# Development of a topical bacteriophage gel targeting *Cutibacterium acnes* for acne prone skin and results of a phase 1 cosmetic randomized clinical trial

**DOI:** 10.1002/ski2.93

**Published:** 2022-01-28

**Authors:** M. Golembo, S. Puttagunta, U. Rappo, E. Weinstock, R. Engelstein, I. Gahali‐Sass, A. Moses, E. Kario, E. Ben‐Dor Cohen, J. Nicenboim, H. Ben David, K. Sudakov, A. Cohen, M. Bassan, N. B. Zak

**Affiliations:** ^1^ BiomX Ltd Ness Ziona Israel; ^2^ BiomX Inc Branford Connecticut USA

## Abstract

**Background:**

Topical antibiotics are frequently used to treat acne vulgaris. Their prolonged use, often for longer durations than recommended, has led to antibiotic resistance in *Cutibacterium acnes (C. acnes)*, a bacterium implicated in acne pathophysiology. Bacteriophage (phage), which specifically target *C. acnes* by a different mechanism of action and do not harm potentially beneficial bacteria, may offer an alternative approach for improvement of the appearance of acne prone skin.

**Objectives:**

To identify and characterize *C. acnes* targeting phage, carry out a comprehensive preclinical safety evaluation of phages selected for further development and examine their safety, tolerability and ability to target facial *C. acnes* when applied topically in a cosmetic clinical study including participants with mild‐to‐moderate acne.

**Methods:**

Phages were isolated by conventional microbiological methods also used to examine their breadth of host range on different *C. acnes* strains and specificity to this bacterial species. Safety assessment of three selected phages was carried out by complete genomic analysis to assure the absence of undesired sequences and by ex vivo models employed to evaluate the safety, irritability and potential systemic bioavailability of phage applied topically. A randomized, controlled clinical study assessed safety, tolerability and efficacy in targeting facial *C. acnes*.

**Results:**

Wide host range phages that also target antibiotic resistant *C. acnes* were identified. Their genomes were shown to be free of undesired genes. The three‐phage cocktail, BX001, was not irritant to human skin or ocular tissues in ex vivo models and did not permeate through human epidermis. In a cosmetic clinical study, topically applied BX001 was safe and well tolerated and reduced the facial burden of *C. acnes*.

**Conclusions:**

Combined in silico and ex vivo approaches successfully predicted the observed safety and efficacy of *C. acnes* targeting phage when these were topically administered in a well‐controlled cosmetic clinical study.

## Graphical abstract

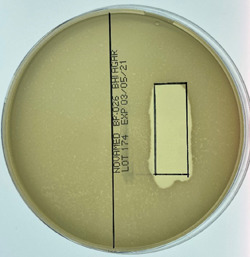

1

**What is already known about this topic?**

- *Cutibacterium acnes* are implicated in the aetiology of acne vulgaris.
- Topical antibiotics are commonly used in the treatment of this condition.
- The efficacy of antibiotics in treatment of acne is decreasing in correlation with increased antibiotic resistance of *C. acnes*.
- For this reason, and in the interest of antibiotic stewardship, especially among the younger population, there is a need for alternative solutions for acne prone skin.

**What does this study add?**

- Bacteriophage that specifically target *C. acnes* are combined in BX001 and formulated in a topical gel.
- Multiple aspects of safety of BX001 are demonstrated by comprehensive preclinical in silico, ex vivo and in vitro approaches.
- BX001 safety and tolerability are confirmed in a double‐blind, randomized, vehicle‐controlled cosmetic trial.
- BX001 is shown to significantly reduce facial *C. acnes* compared to vehicle.

**What is the translational message?**

- Phage cocktails can be designed to target a wide host range of *C. acnes,* including antibiotic resistant strains.
- Safety of phage preparations for topical use can be assessed using in silico and ex vivo methods.
- Topically applied formulated phages are safe and well tolerated when administered to subjects with mild‐to‐moderate acne vulgaris.
- By reducing *C. acnes*, phages potentially offer a microbiome modulating option for acne prone skin.

## 2 INTRODUCTION

Acne vulgaris has been linked to dysbiosis in the skin microbiome.^1^ *Cutibacterium acnes* (formerly *Propionibacterium acnes*), the predominant facial skin bacteria, is thought to play a critical role in the aetiology of acne vulgaris.^2^ Acne is a chronic inflammatory condition characterized by multiple pathogenic factors that bring about a perturbation of the balance of the skin microbiome. Excessive sebum secretion in response to hormonal triggers and clogging of hair follicles due to hyper‐keratinization both create optimal conditions for proliferation of the sebum‐dependent *C. acnes* and subsequent *C. acnes* triggered inflammation of the pilosebaceous units. Recent studies suggest that some *C. acnes* strains may be more frequently associated with normal skin and others with acne.^1^, ^3^

Use of antibiotics, both topical and oral, is a mainstay of acne treatment. The prolonged use and overuse of antibiotics has led to extensive antibiotic resistance in *C. acnes* strains.^4^, ^5^, ^6^ Globally, *C. acnes* resistance to antimicrobials has increased almost 40% between the 1980s and 2000s,^7^ making antibiotic treatment progressively less effective and antibiotic resistance one of the main causes of treatment failure in acne vulgaris.^8^ Therefore, alternative approaches are being sought to address acne prone skin by reducing the pathological bacterial component, preferably without affecting beneficial commensal skin bacteria.

A recent analysis of the skin microbiome in healthy individuals, individuals with acne and older individuals, revealed a significantly increased prevalence and abundance of *C. acnes* bacteriophages (phages) in the healthy group compared to individuals with acne (*p* = 0.05), thus linking phage abundance to healthy skin.^1^ A positive correlation between *C. acnes* phage abundance and subject age was also observed, suggesting that a lower prevalence of acne in older individuals may be due to increased abundance of *C. acnes* phage, in addition to changes in the cutaneous ecological niche, such as reduced sebum. These findings suggest a role for *C. acnes* phages in acne‐free skin, potentially by modulating the *C. acnes* populations.^1^, ^3^

Phages are bacterial species‐specific and strain‐limited viruses that are considered inert to mammalian cells.^9^ The self‐amplifying nature of strictly lytic phages (that cannot integrate into their target bacterial genome) assist effective levels to be achieved locally. Their replication is self‐limiting; once target host bacteria are significantly diminished, phages are unable to replicate and are eliminated from the body.^10^ Many phages are able to penetrate biofilm, a common feature of bacterial growth that confers an additional level of antibiotic resistance^11^ and has been proposed to play a role in pathogenesis of acne vulgaris.^12^ Phages thus offer a natural treatment method to beneficially alter microbiome composition. They are often administered as ‘cocktails’ comprised of individual phages with different targeting characteristics to address all relevant strains and reduce the risk of emergence of phage resistance.

The strong safety profile of natural phages is supported by their ubiquity, specificity to bacterial subsets,^9^ long history of use in the former Soviet Union and Eastern Europe,^13^ well documented cases of compassionate use without severe adverse events (AEs)^14^, ^15^ and the results of early phase clinical trials recently initiated in the West.^16^ Some phages have obtained regulatory recognition as Generally Regarded as Safe for use in food and plant protection^17^ and the FDA has expressed that conventional Good Laboratory Practice (GLP) toxicology studies in healthy animals are not required prior to initial administration to humans.^18^

Phages that target *C. acnes* have been described and their potential to improve the appearance of acne prone skin suggested.^6^, ^19^ However, to date there has been no reported safety assessment carried out on phage preparations as a step towards development of a natural, commercially viable, phage‐based alternative for acne‐prone skin. We describe the characteristics and activity of three novel, newly isolated, *C. acnes‐*specific natural bacteriophages used to design a phage cocktail which is also active on antibiotic‐resistant and biofilm embedded bacteria. We outline the various in silico and experimental approaches taken to demonstrate the safety of the individual phages and of the phage cocktail as an essential step towards further development. Finally, we describe the results of a first‐in‐human randomized, double‐blind, vehicle‐controlled cosmetic study evaluating the safety, tolerability and effect on facial *C. acnes* of a topical cosmetic gel containing the BX001 cocktail in subjects with mild‐to‐moderate acne.

## 3 MATERIALS AND METHODS

Details are described in Supporting Information (Appendix S1—S4).

## 4 RESULTS

### 4.1 Phage isolation and design of BX001 cocktail

**FIGURE 1:**
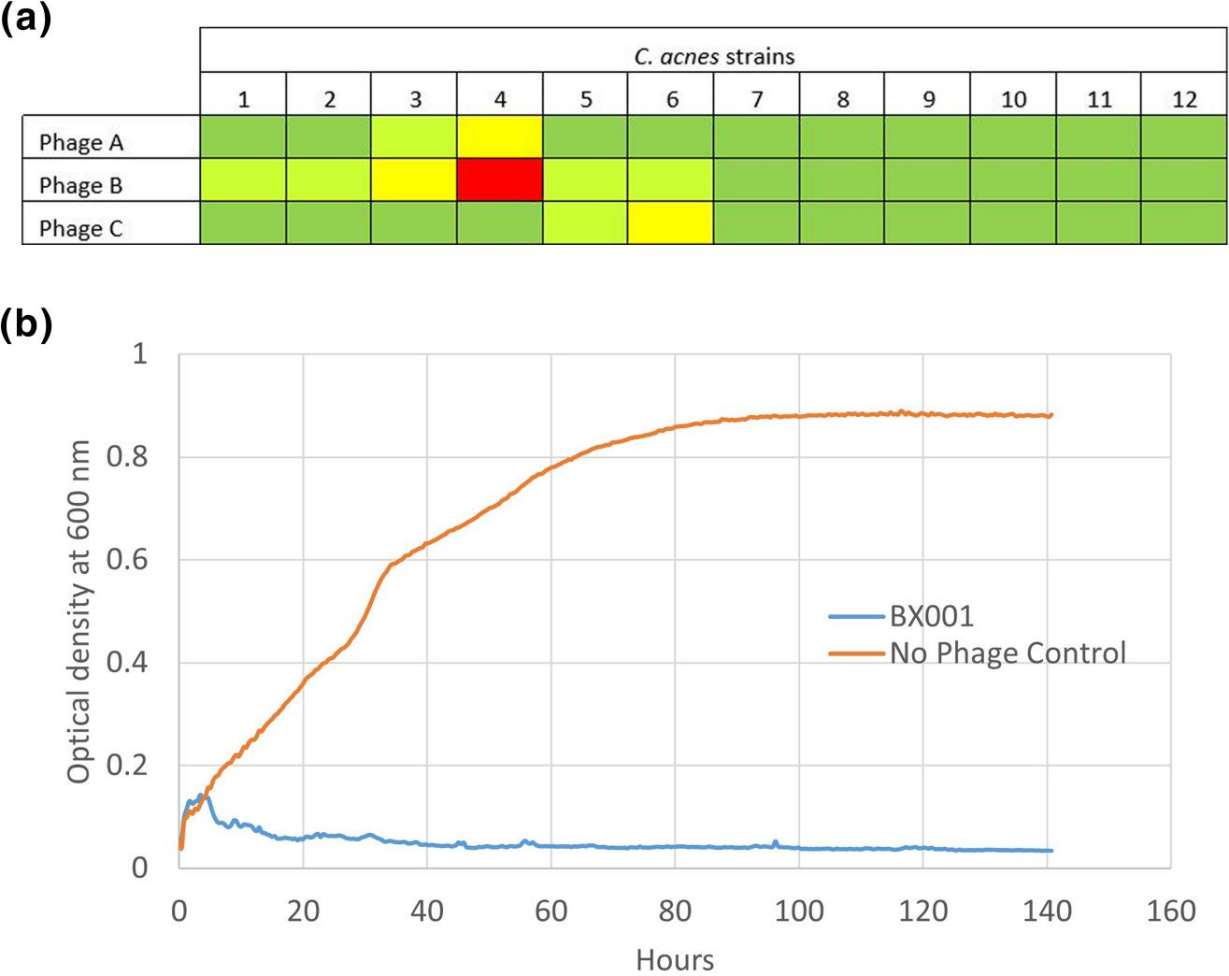
Host range and activity of BX001 bacteriophages. (a) Host range of each of the three selected phages in BX001 on a panel of 12 commercially and internally sourced *C. acnes* strains. Testing was carried out in triplicate by double layer agar spotting assay (see Appendix S1 in the Supporting Information). Dark green indicates total clearing of the spot or a number of plaques too numerous to count, light green indicates more than 10 plaques, yellow indicates between 1 and 10 plaques and red indicates that no plaques (no infectivity) were observed. (b) Sustained inhibition of *C. acnes* growth by BX001 cocktail (blue curve) as measured by OD (OD600) in a liquid culture at 37°C, compared to untreated bacterial growth (orange curve). OD600 measurements were carried out every 15 min in a Tecan Infinite M200 plate reader connected to a Tecan EVO75 robot. No spike in bacterial OD that would indicate the emergence of phage resistant bacteria was observed in the presence of BX001 phages over a period of 140 h. These results strongly suggest that the possibility of the emergence of a viable phage resistant bacterial strain upon administration of the BX001 cocktail has been greatly mitigated by design of a cocktail with phages originating from different environmental samples, and thus unlikely to occur together in nature, and possessing complementary characteristics indicating that they may be acting through different receptors

A total of 21 phages which target *C. acnes* were isolated as described in Appendix S1 (see Supporting Information) and 3 were selected for inclusion in the phage cocktail termed BX001 based on their wide, diverse and complementary patterns of infectivity on an initial representative panel of 12 *C. acnes* strains (Figure 1a). To minimize the potential for development of phage resistance, the cocktail was designed so that each representative target bacterial strain was susceptible to more than a single phage (Figure 1a). BX001 displayed 96% infectivity on 119 clinical strains isolated from subjects with and without acne, including antibiotic‐resistant and acne‐associated strains (see Table S1 in the Supporting Information). When introduced to liquid *C. acnes* cultures, BX001 efficiently prevented bacterial growth, observed as a sustained decrease in optical density (OD) of the culture medium compared to the OD of bacteria alone, over an extended period of time (140 h; Figure 1b).

The selected phages were further shown to have high specificity for *C. acnes* without susceptibility of other commensal and potentially beneficial skin species (see Table S2 in the Supporting Information) and to be active against *C. acnes* bacteria embedded in biofilm (see Appendix S1 and Figure S1 in the Supporting Information, for methods and calculations^20^, ^21^, ^22^), supporting their suitability for activity in the skin environment.

### 4.2 Activity of BX001 cocktail formulated in an aqueous gel for topical application

The BX001 phage cocktail was formulated into an aqueous hydroxyethylcellulose (HEC) gel (Natrosol 250 HHX) for topical application and examined for bacteriophage release from this matrix allowing subsequent activity on a *C. acnes* lawn and in an ex vivo model of reconstituted human skin colonized with *C. acnes* (see Appendix S2 in the Supporting Information).

**FIGURE 2:**
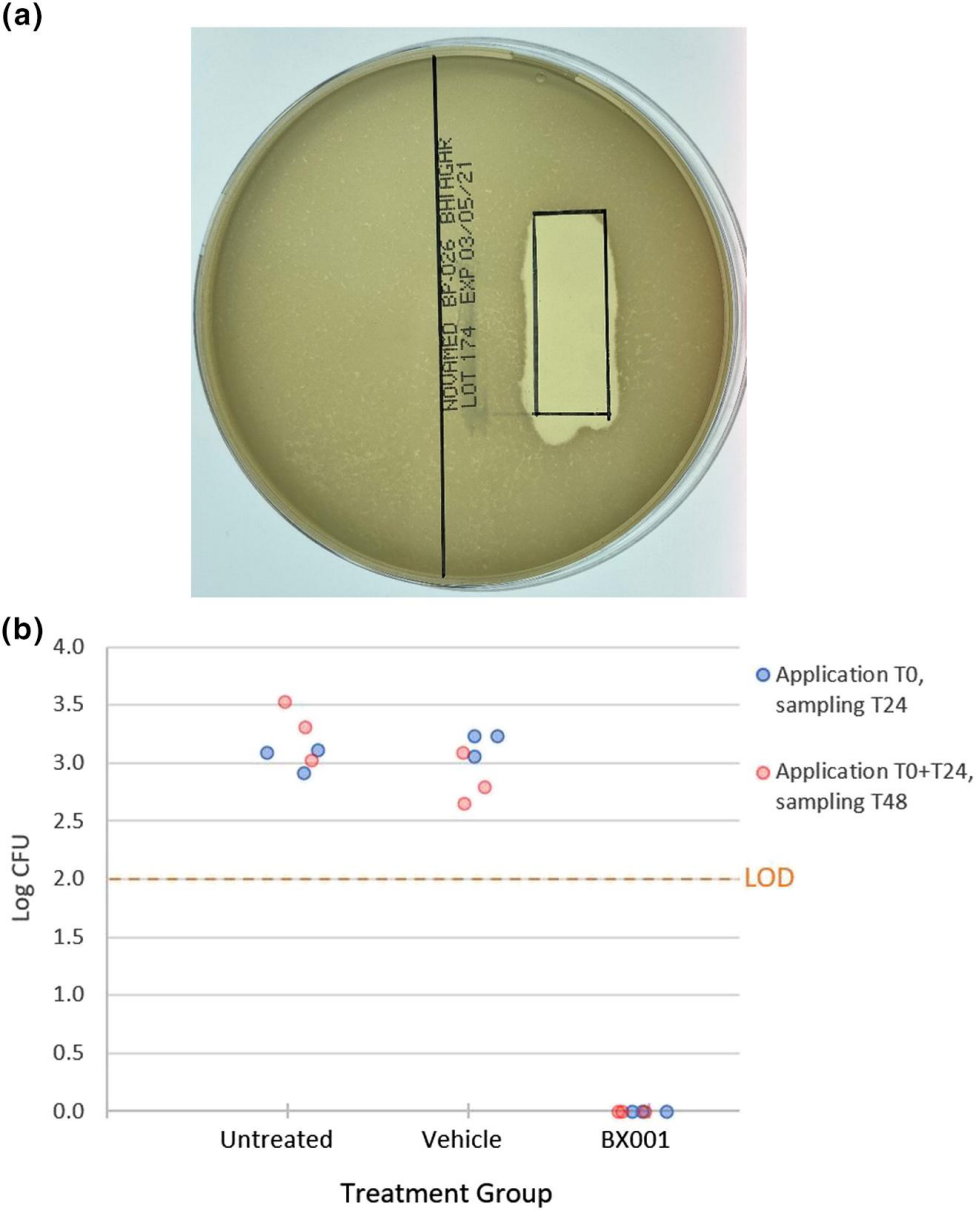
Activity of BX001 bacteriophages in formulated aqueous gel. (a) Formulated BX001 gel with high dose bacteriophage was applied to a pre‐marked region on the bacterial lawn and incubated anaerobically at 37°C overnight. The clear area demonstrates killing of the underlying bacteria by the phages within the gel. (b) Formulated BX001 gel was applied onto reconstituted artificial skin colonized with *C. acnes* NCTC 737 (isolated from an acne lesion on human facial skin). Skin inserts were inoculated with 1 × 10^6^ Colony Forming Units (CFU)/cm^2^ of *C. acnes* NCTC 737 and incubated at 37°C for 1 h to dry. About 10 μL of BX001 gel (high dose) or vehicle gel (placebo) were applied to three inserts each at *t* = 0 and to another set of three inserts each at *t* = 0 and again at *t* = 24 h. Six inserts were left untreated as controls of *C. acnes* growth at two timepoints. At 24‐ and 48‐h post‐test item administration, inserts were assessed for bacterial burden. The graph shows log_10_ of *C. acnes* CFU recovered. In all BX001 treated skin inserts bacterial burden was below the Limit of Detection (LOD) suggesting a reduction by at least 90% compared to untreated or vehicle treated inserts. A statistical analysis using *t*‐test for two samples assuming unequal variances and testing the hypotheses that both means are equal and *α* = 0.05 (with absence of CFU counting as zero) yielded a significant result for BX001 treated inserts compared to untreated at both 24 and 48 h (*p* = 0.0078 and 0.0413, respectively) whilst the comparison of vehicle to untreated did not display significance at either timepoint (*p* = 0.0862 and 0.0795)

Clearing of the opaque bacterial lawn directly below where the gel was applied (black square, Figure 2a) demonstrates release of phage from the HEC gel and lysis of the underlying target bacteria. No clearing was observed with the gel alone (not shown).

Figure 2b shows results after application to full thickness reconstituted human skin model multiwell inserts comprised of fully differentiated dermal and epidermal components (Labskin) which were colonized with *C. acnes*. Formulated BX001 or vehicle were then applied to six inserts each. An additional dose was applied to three inserts from each group after 24 h. *C. acnes* bacterial burden was measured 24 h after the last application to each group. A significant difference was observed in *C. acnes* load between inserts treated with BX001 phage containing gel and vehicle following single and repeat applications (*p* = 0.0078 and = 0.0413, respectively), whilst the vehicle samples did not differ from untreated inserts.

### 4.3 In silico safety assessment of individual phages

As more phage applications are being developed, Western regulatory agencies have clarified the intrinsic properties required of phages intended for human administration.^23^ These comprise a strictly lytic lifecycle, absence of sequences encoding undesirable genes and lack of generalized transduction potential.

A proprietary automated bioinformatics platform was established to examine phage sequences for the presence of undesirable genes. These include integrase genes indicative of phages’ ability to insert their genome into that of the host, all known toxins specified in 40 C.F.R. §725.421,^23^ virulence genes,^24^ antibiotic resistance genes^25^ and bacterial host sequences indicative of phage potential to transduce genomic material from one host to another (see Appendix S3 in the Supporting Information).

The coding sequences of each of the three phages comprising BX001 were aligned by BLAST^26^ against all sequences of this proprietary platform and demonstrated to be free of the above detailed sequences, thus supporting a high degree of intrinsic safety.

### 4.4 Ex vivo safety assessment of BX001 phage cocktail on reconstituted human tissues

The safety of BX001 phage preparations was assessed under GLP using OECD (Organization for Economic Co‐operation and Development) approved ex‐vivo systems commonly employed for safety evaluation of topical products.^27^, ^28^ These comprised a skin irritation test following the OECD TG (test guideline) 439, using reconstituted human epidermis (EpiDerm™ MatTek), and an eye irritation test following the OECD TG 492, using reconstituted human corneal‐like epithelium (EpiOcular™ MatTek).

EpiDerm™ tissues were exposed to three concentrations of BX001 phages: high, medium and low, representing ∼100‐fold, ∼10‐fold and ∼1‐fold the maximal intended amounts per square centimetre for skin application, in parallel to a sodium magnesium (SM) buffer control (vehicle), a positive control (5% sodium dodecyl sulphate solution, SDS) and a negative control (Dulbecco’s phosphate buffered saline rinse solution).

**FIGURE 3:**
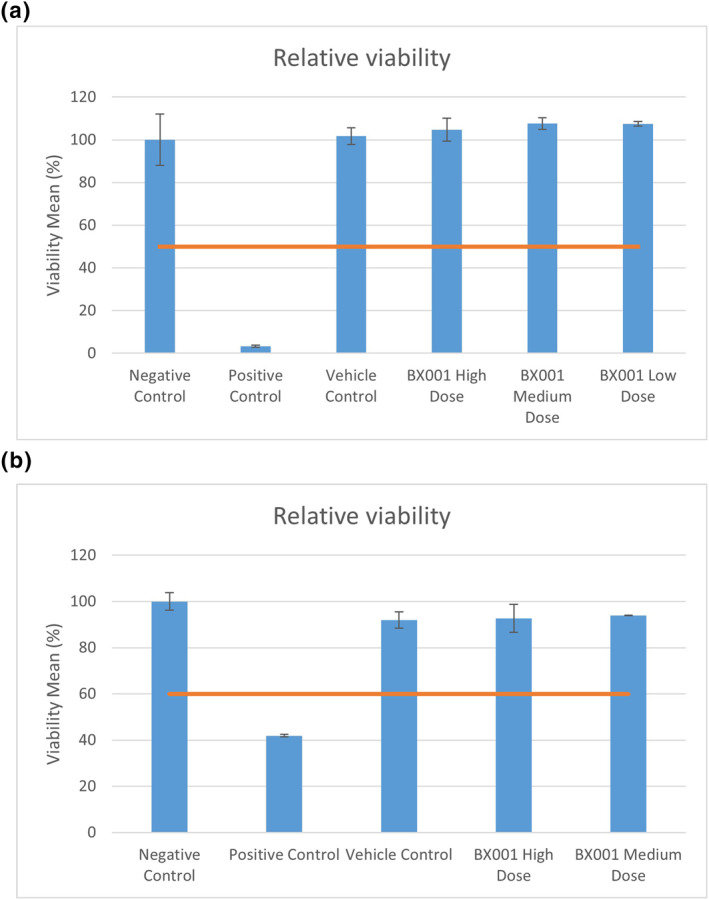
Testing for irritation potential by relative viability after application of BX001 phages to reconstituted human skin and ocular tissue models. (a) Reconstructed human epidermal tissues (EpiDerm™ MatTek) consisting of normal human‐derived epidermal keratinocytes were exposed for 35 min to BX001 phages at 100× (high), 10× (medium) and 1× (low) fold concentrations of the maximal intended dose for human exposure and compared to tissues exposed to negative control (DPBS Rinse Solution, #TC‐PBS, MatTek Corporation), positive control (5% SDS Solution, #TC‐SDS‐1, MatTek Corporation) and vehicle control (SM buffer) with respect to relative viability. Cell viability was measured by MTT [(3‐4,5‐dimethyl thiazole 2‐yl) 2,5‐diphenyltetrazoliumbromide] assay performed per manufacturer’s instructions and with reagents provided by the manufacturer. None of the test items yielded values below 50% relative viability (red line) and they are considered non‐irritants. (b) Reconstructed human cornea‐like epithelium tissues (EpiOcular™ MatTek) were exposed in triplicate to BX001 phages at high (100×) and medium (10×) concentrations and compared to negative control (tissue culture grade water, #03‐055‐1A, Biological Industries), positive control (Methyl acetate, #TC‐MA, MatTek Corporation) and vehicle control (SM Buffer) with respect to relative viability (%) as above. None of the test items yielded results below 60% relative viability (red line) and they are considered non‐irritants. Data represent mean ± SD (standard deviation)

**FIGURE 4:**
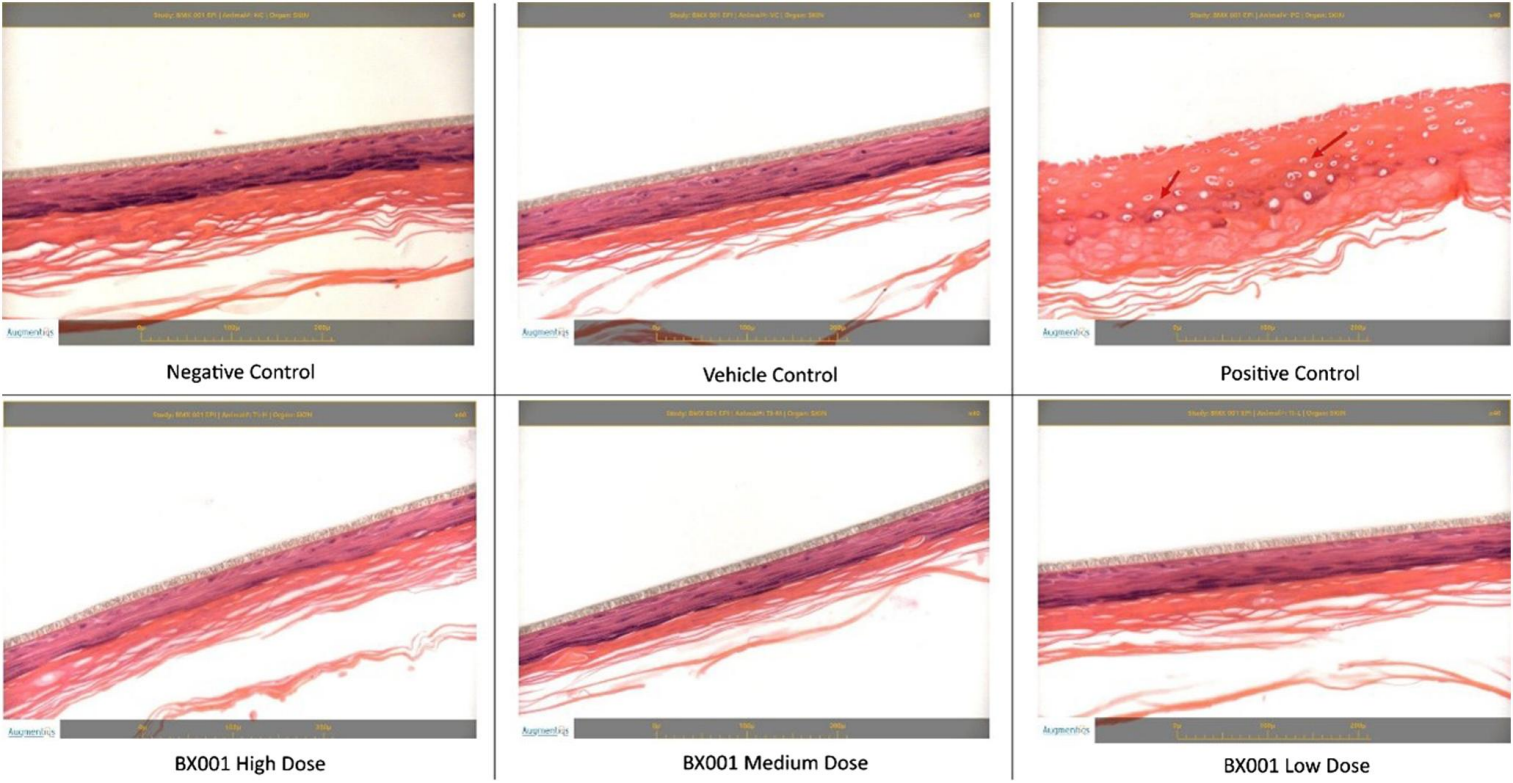
Histopathological evaluation of reconstituted human epithelial tissues exposed to BX001 phages at different doses. Tissues were sectioned at approximately 5 μm thickness and stained with haematoxylin and eosin. No treatment‐related changes were seen in the negative control, vehicle control or any sample exposed to the BX001 phage preparations. Diffuse epidermal necrosis was observed in the positive control (red arrows)

Following exposure, triplicate samples from each test group were monitored for viability using a colorimetric MTT [(3‐4,5‐dimethyl thiazole 2‐yl) 2,5‐diphenyltetrazoliumbromide] assay which assesses cell metabolic activity. An additional representative tissue was sectioned for histologic evaluation. The mean viabilities of tissues exposed to high, medium and low phage concentrations were 104.8%, 107.6% and 107.5%, respectively, compared to the negative control. A similar result was obtained for the vehicle control (101.7%) whilst the positive control showed 3.4% viability. Thus, no irritation effect, defined as viability reduction to ≤50% as per OECD TG 439, was indicated for the phage preparations at any concentration (Figure 3a). No treatment‐related histological or inflammatory changes were observed after phage exposure at any concentration (Figure 4).

Similarly, EpiOcular™^20^ tissues were exposed in triplicate to high and medium concentrations of BX001 phages in parallel to vehicle, positive and negative controls. The mean viabilities of exposed tissues were 92.7% and 94.0% for high and medium doses respectively, comparable to a mean viability of 92.0% for the vehicle control. Thus, there was no irritation effect (defined as viability ≤60%) for either dose, whilst the positive control displayed 41.9% viability, indicative of a significant irritation effect.

These studies using reconstructed human tissues demonstrate that BX001 phage cocktail is non‐irritant to skin or corneal epithelia even at the highest concentration tested (representing ∼100 fold of maximal intended dose for human exposure).

### 4.5 Evaluation of BX001 phages permeation through epidermis

The potential of topically applied BX001 phages to permeate through the epidermis into the vascularized dermal layer of human skin was examined by a GLP ex vivo skin permeation experiment using a human epidermal layer in a Franz cell setup (see Appendix S3 in the Supporting Information). The apical surface of the epidermal tissues (*n* = 6) was exposed to BX001 phages at the highest concentration intended for topical application and permeation of phages into the underlying receiving chamber was measured by plaque assay after 24 h. Low levels of phage were detected in the receptor compartment in three of the six tested epidermal samples, resulting in an average of 0.0039% of the total applied amount. The results indicate a very low level of permeation through the epidermal layer, suggesting negligible systemic availability.

### 4.6 Phase 1 clinical trial of BX001 gel in subjects with mild‐to‐moderate acne

Following the above safety assessment and manufacture of BX001 according to Good Manufacturing Practice, a clinical study evaluated the safety and efficacy of BX001 topical gel on acne‐prone skin (see Appendix S4 in the Supporting Information). A total of 75 participants with mild‐to‐moderate acne vulgaris were randomized in a double‐blinded fashion to one of three cohorts with once daily application to the face of a high dose, a 2 log_10_ lower dose or vehicle gel for 4 weeks with a 1‐week follow‐up. Baseline characteristics were similar across all cohorts (see Table S3 in the Supporting Information).

Assessment of AEs by treatment group showed low rates of treatment‐related AEs (see Table S4 in the Supporting Information). All AEs were mild or moderate; there were no serious AEs, and no AEs leading to premature discontinuation.

Both doses of BX001 demonstrated excellent tolerability profiles. Frequency distributions of the Investigator’s Assessment of tolerability by treatment group at the End of Treatment (Day 28) are shown in the Table S5 of the Supporting Information.

### 4.7 Efficacy analyses

**FIGURE 5:**
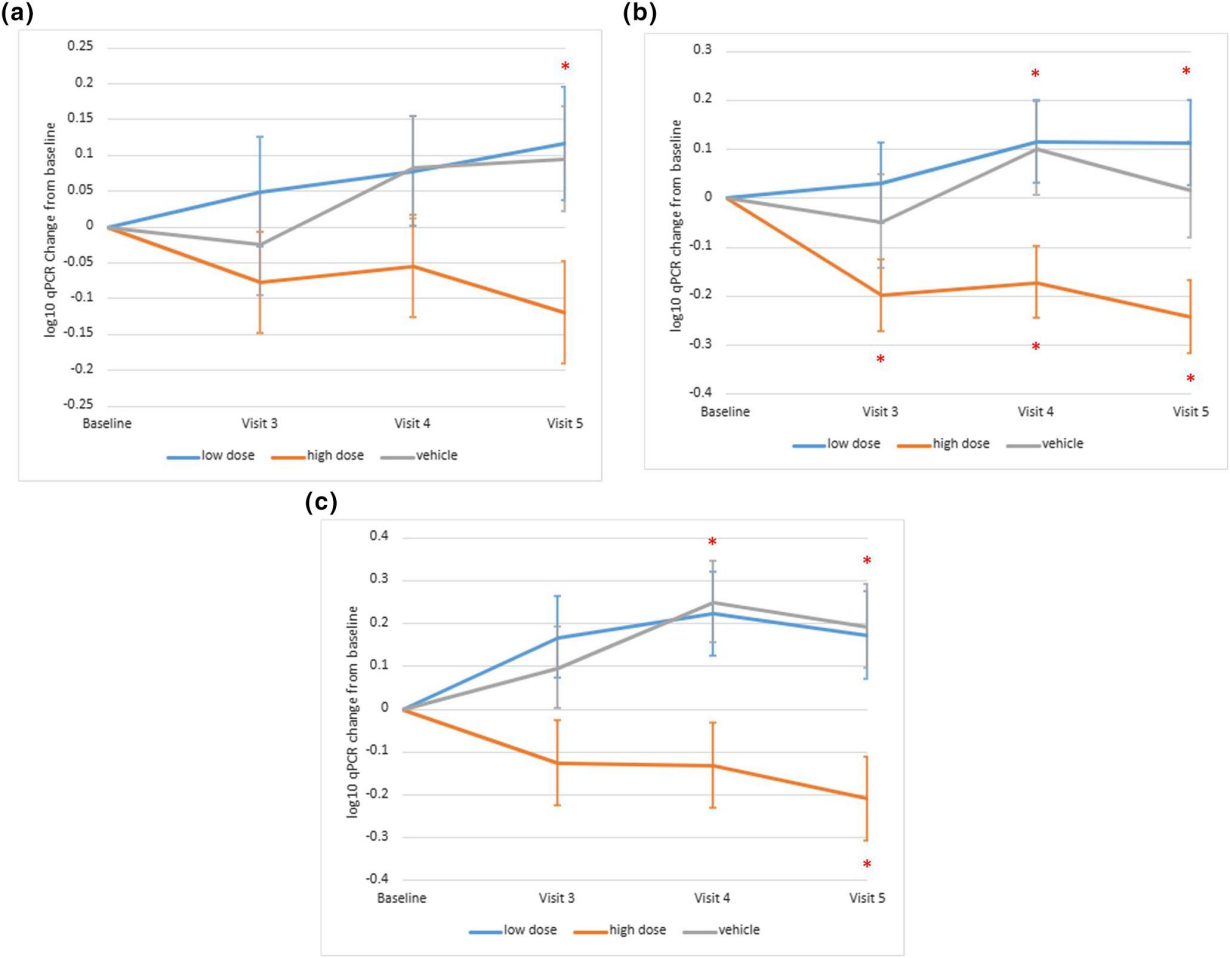
Plots of mean changes from baseline in (a) *Cutibacterium* spp. qPCR log_10_ (copies/sample) over time (Per Protocol population), (b) qPCR response by baseline *Cutibacterium* spp. bacterial burden (Full Analysis set) and (c) qPCR response by baseline sebum (Full Analysis set). Asterisk indicates a statistically significant difference between the BX001 high dose group and vehicle (upper asterisk) or within BX001 high dose group compared to baseline value (lower asterisk). Data represent mean ± SEM (standard error of the mean)

Efficacy of BX001 in reducing bacterial load was assessed by quantifying *Cutibacterium* species (spp) using a *Cutibacterium* spp. specific qPCR over time and relative to vehicle (Appendix S4 in the Supporting Information). The species specific qPCR is adequate as *C acnes* constitutes >85% of skin microbiota and other *Cutibacteria* constitute only up to 2.5%^1^, ^3^ Subjects in the high dose group had a sustained reduction in mean *Cutibacterium* spp. levels compared to baseline (Visit 2), starting at Day 14 (Visit 3) and through Day 35 (Visit 5, 1 week after last dose) with a statistically significant reduction of −0.22 logs at Day 35 compared to vehicle (*p* = 0.036). On Day 35 a 0.12 log reduction, which translates to a 24% reduction in *C. acnes* levels, was observed in the high dose cohort compared to baseline, whilst a 0.1 log increase, which translates to a 26% increase from baseline, was observed in the vehicle cohort. The lower dose did not bring about reduction in target *Cutibacterium* load (Figure 5a).

Subgroup analyses assessed drivers of efficacy, with cutoffs based on median values of relevant characteristics at baseline (Figure S2 in the Supporting Information). Subjects with higher baseline bacterial burden (>5.95 log) or baseline sebum (>133 μg/cm^2^) had a more pronounced reduction in *Cutibacterium* levels after treatment with high dose BX001.

In the subgroup with a baseline *Cutibacterium* level >5.95 log_10_, a significant reduction of bacterial load was observed in the BX001 high dose group compared to vehicle at Visits 4 (Day 28) and 5 (Day 35) and to baseline at all visits (Figure 5b). No significant reduction was observed in the low bacterial burden subgroup (see Figure S2a in the Supporting Information).

In the subgroup with a baseline sebum >133 μg/cm,^2^ a significant *Cutibacterium* reduction was observed in the BX001 high dose group compared to vehicle at Visits 4 (Day 28) and 5 (Day 35) and to baseline at Visit 5 (Day 35; Figure 5c). This was not observed in the low sebum level subgroup (see Figure S2b in the Supporting Information).

**FIGURE 6:**
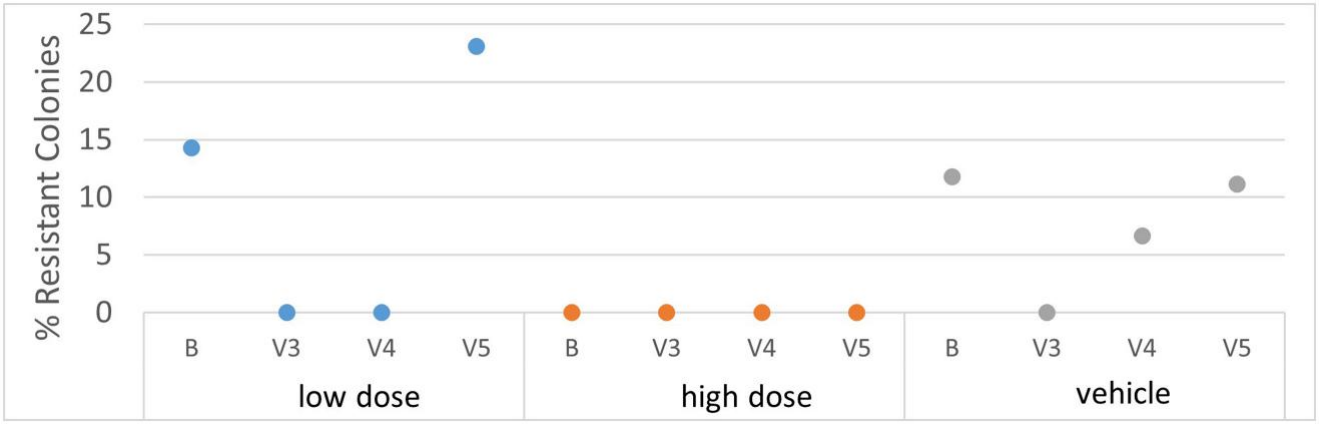
Sensitivity of facial *Cutibacterium acnes* to BX001 following exposure to BX001 or vehicle. *C. acnes* identity was confirmed by MALDI‐TOF analysis. Resistance at baseline (B, prior to exposure to BX001) was 14% (*N* = 14) for the low BX001 group, 0% (*N* = 14) for the high BX001 group and 12% (*N* = 17) for the vehicle group. At 14 days of treatment (V3), resistance was 0% for all groups (*N* = 15 in low BX001 group, *N* = 13 in high BX001 group and *N* = 16 in vehicle group). At 28 days of treatment (V4) resistance was 0% for low and high groups (*N* = 19 and 17, respectively), and 7% for the vehicle group (*N* = 15). One week after the last application of the test items (V5) resistance was 23%, 0% and 11%, respectively, for the three groups (*N* = 13, 14 and 18). The average overall resistance for all isolates collected at all timepoints was 5%, similar to what was observed during preclinical testing (see Table S1 in the Supporting Information). B = Baseline; *V*3 = 14 days, *V*4 = 28 days, *V*5 = 35 days or 1 week after last application

The extent of phage susceptibility in the facial *C. acnes* of subjects at baseline and the emergence of phage resistance over the course of the study were examined. A total of 13–19 *C. acnes* isolates from subjects who received high dose BX001, low dose BX001 or vehicle, were collected at baseline (Visit 2) and three additional timepoints and examined by plaque assay for BX001 sensitivity. At baseline 4 out of 45 (9%) of all the isolates were resistant to BX001. Similar levels were maintained by all study groups over time (Figure 6), supporting the notion that repeated exposure to BX001 is not associated with development of phage resistance.

## 5 DISCUSSION

The wide host range of individual *C. acnes* phages, each of which targets many *C. acnes* strains, has allowed the development of BX001, an efficient cocktail with a minimal number of component phages. The killing capability of BX001 phages on *C. acnes* in solution, on a bacterial lawn and in the context of colonized reconstituted human skin, together with the specificity for *C. acnes* and the demonstration that BX001 phages are active against antibiotic resistant *C. acnes* strains and bacteria embedded in biofilm, support the potential use of BX001 in modulating and rebalancing the skin microbiome of subjects with acne prone skin.

Safety of BX001 for human topical application was established utilizing a variety of methods which include an automated bioinformatic platform incorporating information from multiple databases to demonstrate the intrinsic safety of the individual phage based on sequence analysis and well‐recognized ex vivo models to determine the lack of irritation and minimal penetration of phage preparations in relevant human tissues.

The two ex vivo OECD validated assays used under GLP conditions were demonstrated to offer an acceptable alternative to animal models, with the potential of being even more predictive due to their human origin. The systems have been referenced in several internationally accepted OECD test guidelines for assessing the safety of substances for regulatory purposes.^27^, ^28^

Permeation levels of phage through human epidermal tissue were found to be minimal. This was further confirmed in a non‐GLP Franz cell study employing Strat‐M™ (Merck Millipore), a synthetic membrane that mimics the skin barrier, in which no permeation of phage to the receptor chamber was detectable over 24 h (not shown).

Recent clinical cases describing the safe use of high doses of phages administered by various routes, including intravenously^15^, ^29^, ^30^, ^31^ and historical reports of safe applications^32^ along with the preclinical results of this study supported clinical testing of BX001 as a safe microbiome modulating topical gel for acne prone skin through its selective activity on *C. acnes*. The double blind, vehicle‐controlled study with 75 female subjects randomized to three cohorts for once daily application of high dose, low dose or vehicle for 28 days, demonstrated that the BX001 cosmetic product at both doses was very well‐tolerated, with a low AE profile similar to vehicle. All AEs were mild or moderate, and no SAEs were observed. There were no physical exam findings or laboratory test results of clinical concern (see Appendix S5 in the Supporting Information) further supporting the safe use of BX001 as an alternative to current acne treatments. Skin microbiome diversity assessed by the Shannon index with taxonomic assignation using Qiime^33^ did not change significantly in the Per Protocol population or Full Analysis Set after BX001 administration (see Appendix S5 in the Supporting Information).

Efficacy of BX001 in targeting facial *C. acnes* was demonstrated in a dose‐dependent manner. Only subjects in the BX001 high dose group showed a statistically significant reduction in mean change from baseline of *Cutibacterium* levels compared to vehicle, at Day 35. Subgroup analyses revealed that subjects with the related characteristics of oilier skin or higher baseline bacterial load showed a more pronounced reduction in *Cutibacterium* load, possibly due to more frequent target engagement, suggesting that such subjects may benefit most from application of BX001.

There was no evidence of emergence of phage resistant bacteria on facial skin following repeated exposure to BX001, thus confirming efficacy of the cocktail in inhibiting bacterial resistance and supporting its potential extended use.

Studies with longer use of BX001 may elucidate possible benefits of *C. acnes* phage in improving the appearance of acne prone skin.

## CONFLICT OF INTEREST

Myriam Golembo, Sailaja Puttagunta, Urania Rappo, Eyal Weinstock, Inbar Gahali‐Sass, Ayelet Moses, Edith Kario, Eyal Ben‐Dor Cohen, Julian Nicenboim, Ariel Cohen, Merav Bassan and Naomi B. Zak are current employees of BiomX and may own stock. Roni Engelstein, Hava Ben David and Kobi Sudakov were employees of BiomX at the time the research was conducted and may own stock.

## AUTHOR CONTRIBUTIONS

**Myriam Golembo**: Conceptualization; Formal analysis; Investigation; Methodology; Project administration; Writing—original draft; Writing—review and editing. **Sailaja Puttagunta**: Conceptualization; Formal analysis; Investigation; Methodology; Supervision; Writing—original draft; Writing—review and editing. **Urania Rappo**: Formal analysis; Investigation; Writing—original draft; Writing—review and editing. **Eyal Weinstock**: Conceptualization; Data curation; Formal analysis; Investigation; Writing—original draft; Writing—review and editing. **Roni Engelstein**: Data curation; Formal analysis; Investigation; Methodology; Project administration; Writing—original draft; Writing—review and editing. **Inbar Gahali‐Sass**: Formal analysis; Supervision; Writing—review and editing. **Ayelet Moses**: Investigation; Writing—review and editing. **Edith Kario**: Investigation; Writing—review and editing. **Eyal Ben‐Dor Cohen**: Investigation; Writing—review and editing. **Julian Nicenboim**: Investigation; Writing—review and editing. **Hava Ben David**: Investigation; Writing—review and editing. **Kobi Sudakov**: Investigation; Methodology; **Ariel Cohen:** Formal analysis; Investigation; Supervision; Writing—original draft; Writing—review and editing. **Merav Bassan**: Formal analysis; Investigation; Supervision; Writing—original draft; Writing—review and editing. **Naomi Zak**: Conceptualization; Formal analysis; Investigation; Writing—original draft; Writing—review and editing.

## ETHICS STATEMENT

The study was conducted under IRB MHMC‐17‐0032. Written informed consent was obtained from all patients.

## Supporting information

Supporting Information S1

## Data Availability

Data available in article supplementary material.
