# Supplementary material for: Development of a topical bacteriophage gel targeting Cutibacterium acnes for acne prone skin and results of a phase 1 cosmetic randomized clinical trial
Source: Skin Health Dis. 2022 Jan 28;2(2):e93. doi: 10.1002/ski2.93 (PMC9168013; doi:10.1002/ski2.93)
Supplement: Supplementary file 1 — Supporting Information S1 [file SKI2-2-e93-s001.docx]

**Supporting Information**

Additional Supporting Information may be found in the online version of this article at the publisher’s website:

**Appendix S1.** Phage isolation and characterization methods.

**Appendix S2**. Activity of formulated BX001.

**Appendix S3**. Safety evaluation methods.

**Appendix S4**. Study design and methods of phase 1 cosmetic clinical trial of BX001 in mild-to-moderate acne.

**Appendix S5.** Additional results from phase 1 cosmetic clinical trial of BX001 in mild-to-moderate acne.

**Figure S1**. *C. acnes* counts in biofilm after treatment with phages or erythromycin.

**Figure S2.** Subgroup analyses of *Cutibacterium* qPCR response in phase 1 clinical trial: (a) by baseline *Cutibacterium* bacterial burden below median (<5.95 log_10_); (b) by baseline sebum levels below median (<133 µg/cm^2^); (c) by baseline inflammatory lesions above median (>10) and below median (<10); (d) by baseline non-inflammatory lesions above median (>35) and below median (<35).

**Table S1**. Characterization of *C. acnes* isolates obtained from acne vulgaris and healthy individuals showing acne severity by Investigator's Global Assessment (IGA) score (ranging from 0: clear to 4: severe), bacterial ribotype, antibiotic sensitivity and sensitivity to BX001.

**Table S2**. Sensitivity of common skin bacterial species to each phage in BX001 as part of safety evaluation.

**Table S3.** Baseline characteristics in phase 1 clinical trial (FAS Population).

**Table S4.** Adverse events by treatment group in phase 1 clinical trial (Safety Population).

**Table S5.** Tolerability at end of treatment in phase 1 clinical trial (PP Population).

**Appendix S1. Phage isolation and characterization methods.**

**Phage isolation**

Phages were isolated from natural sources by screening filtered and concentrated sewage samples for phage activity against three different *C. acnes* hosts whose identity was verified by sequencing. Phage-containing samples were applied to a bacterial lawn of the target bacteria cultured on BHIS medium and phage were isolated following detection of a clear plaque caused by lysis of the target bacteria. In order to obtain a pure phage, repeat isolations were carried out.

**Host range determination**

Activity of phages was tested by double layer agar spotting assay. After overnight growth under anaerobic conditions in Brain Heart Infusion broth (Becton, Dickinson, New Jersey) supplemented with 5 g/L yeast extract (Acros Organics, Belgium) ("BHIS"), target bacteria were diluted 1:3 in BHIS, cultured for an additional 5.5 hour, collected by centrifugation (at 2000xg for 5 minutes, room temperature) and re-suspended in 200 µL BHIS. 100 µL of the concentrated *C. acnes* cultures was mixed with 4 mL molten 0.4% agar BHIS and applied to selective plates previously solidified anaerobically. Following incubation of plates for 20 minutes at room temperature and 40 minutes at 37°C, 10 µL of each phage sample with 10^6^ plaque forming units (PFU)/mL (determined based on activity on a universal host) were pipetted onto the bacterial lawns. Phage were allowed to absorb for 20 minutes and then plates were incubated inverted at 37°C or 24 hours before plaques were counted.

Twelve strains were initially used for host range determination as shown in the Table below.

| **Strain ID in Figure 1** | **Strain name** | **Bank ID** | **Phylotype** | **SLST cluster** | **RT group** | **Acne skin / healthy skin / both based on ribotype** |
| --- | --- | --- | --- | --- | --- | --- |
| 1 | 417/52 [VPI 0391] | ATCC 11828 | II | K9 | 2 | Both |
| 2 | Derm-CH102 | ATCC 33179 | IA1 | A1 | 1 | Both |
| 3 | 3 | ATCC 29399 | IA1 | A1 | 1 | Both |
| 4 | Internal strain PA4 | - | II | K2 | 6 | Healthy |
| 5 | Internal strain PAP | - | IA2 | F4 | 3 | Both |
| 6 | VPI 9 | ATCC 51277 | IB | H1 | 1 | Both |
| 7 | NCTC 5235 | ATCC 6923 | IA1 | A1 | 1 | Both |
| 8 | NCTC 556 [VPI 4978] | ATCC 6922 | IA1 | A1 | 1 | Both |
| 9 | NCTC 4311 [NCTC 2083a] | ATCC 6921 | IA1 | A1 | 1 | Both |
| 10 | [643-C, NCTC 10390] | ATCC 12930 | II | K2 | 6 | Healthy |
| 11 | NCTC 737 [VPI 0389] | ATCC 6919 | IA1 | A1 | 1 | Both |
| 12 | Gerath [23, C-7, VPI 4979] | ATCC 11827 | IA1 | A1 | 1 | Both |

In order to validate the broad host range of the BX001 cocktail on a wide range of clinical *C. acnes* strains, clinical *C. acnes* strains were isolated from healthy adults and individuals with acne of differing severity by plating skin swab extracts on *Cutibacterium* selective BHIS medium containing 20μg/ml furazolidone. These are proprietary BiomX isolates which were characterized with respect to ribotype, as shown in Table S1. Individual colonies were expanded and examined for susceptibility to BX001 as described above

Activity of phages on other skin bacterial species was determined in a similar manner.

To determine antibiotic sensitivity of clinical strains these were plated on antibiotic containing plates as follows.

- BHIS+20µg/mL Furazolidone + 0.5µg/mL clindamycin
- BHIS+20µg/mL Furazolidone + 0.5µg/mL erythromycin
- BHIS+20µg/mL Furazolidone + 5µg/mL tetracycline
- BHIS+20µg/mL Furazolidone + 5µg/mL minocycline.

All subjects signed informed consent and the study was conducted under IRB MHMC-17-0032.

**Activity of BX001 phages in liquid culture**

Bacteria were cultured anaerobically overnight, diluted 1:10 in BHIS, grown until an OD_600_ of about 0.4-0.8 was achieved and then diluted further to a starting OD_600_ between 0.05 and 0.2 OD_6oo_. A volume of 200 µL of culture was dispensed in triplicates into a Nunclon flat-bottomed 96-well plate and mixed with 10 µL of BX001 phage cocktail at a concentration of 10^6^ PFU/mL or 10 µL of BHIS as control. The wells were covered with 50 µL of mineral oil to limit evaporation and a thin sterile optically transparent polyester film to maintain sterility and limit oxygen exposure. OD_600_ measurements were carried out every 15 minutes in a Tecan Infinite M200 plate reader connected to a Tecan EVO75 robot. Between measurements, the plate was incubated with shaking at 37°C.

**BX001 activity on biofilm generated by *C. acnes***

Biofilm was created by culturing *C. acnes* strain ATCC 11828 anaerobically. This strain belongs to the type II phylotype, SLST cluster K9 and ribotype group 2. It was originally isolated from a subcutaneous abscess, but strains of this SLST cluster are readily isolated from skin samples of individuals belonging to both healthy and acne groups. The strain was found to be a strong biofilm producer and the biofilm was a single organism biofilm.

Strain ATCC 11828 was cultured without agitation in the presence of medium (Brain Heart Infusion supplemented with 0.5% yeast extract and 0.05% L-Cysteine) plus 0.5% glucose in a 96 well plate that was sealed (using 8-Well Strip Caps for Thermo-Scientific 96-Well Plates). After 72 hours, when mature biofilm could be observed on the surfaces of the wells, 100 µL of medium was carefully removed (without disturbing the biofilm) and 100 µL fresh medium + 0.5% glucose was added. Four hours later, 10 µL of the respective phage cocktail was added to the corresponding six wells (3 for harvesting at 24 hours and 3 for harvesting at 48 hours) for a final phage concentration of 10^7^ plaque forming units (PFU)/mL, as well as 2 µL of erythromycin in the corresponding six wells (final concentration 100 µg / mL). The sensitivity of *C. acnes* in the biofilm to antibiotics or phage cocktails was evaluated at 24- and 48-hours post-treatment by extracting viable bacteria from sonicated and centrifuged biofilm and documenting the serial dilutions where colonies emerged versus those where no colonies were observed.

Data analysis followed the Most Probable Number (MPN) guidelines in the FDA’s Bacteriological Analytical Manual^27^ using Thomas’ formula^28^ to estimate the MPN; the approximation of the standard error was calculated according to Haldane’s approximation^29^. As described, biofilms, which constitute a common growth pattern of *C. acnes* and other bacteria, are complex structures consisting of bacterial colonies adhering to surfaces that secrete a mucilaginous protective coating in which they are encased. As biofilms are commonly considered to be resistant to antibiotics, the sensitivity of *C. acnes* growing in biofilm to phages was evaluated and compared to the activity of antibiotics. Figure S1 (Supporting Information) shows that, after *C. acnes* biofilm formation and 24 hours of phage treatment, a 4 log (10^4^) decrease in *C. acnes* bacterial counts is observed in all phage-treated arms relative to the untreated control. An additional log decrease was observed after an additional 24 hours of treatment. In contrast, the effect of erythromycin on bacteria growing in biofilm was observed only after 48 hours of treatment as a ~1 log reduction from baseline.

These results demonstrate the efficacy of phages against target *C. acnes* bacteria even when these are growing as within a biofilm. The different phage cocktails had similar strong activity against biofilm, suggesting that a robust biofilm penetrating ability is common across *C. acnes* phages. By comparison, the antibiotic erythromycin, to which this bacterial strain is sensitive, was significantly less effective in lysing *C. acnes* bacteria growing as biofilm and achieves only a one log reduction in viable bacteria after 48 hours of exposure.

**Appendix S2. Activity of formulated BX001.**

**Activity of formulated BX001 phages on bacterial lawn**

A 4 mL *C. acnes* culture was grown overnight anaerobically at 37°C, then spun down (2000xg, 5 min, room temperature). After discarding the supernatant and resuspending in 1 mL of fresh BHIS, 150 µL were mixed with 4 mL of molten 0.4% agar BHIS and poured onto a 1.5% agar BHIS plate. After a 20 min recovery in the biological hood, 10 µL of BX001 gel (high dose) were applied to a pre-marked region on the plate which was incubated anaerobically at 37°C overnight prior to examination.

**Activity of formulated BX001 phages on *ex -vivo* model of infected reconstituted human skin**

The study was carried out at Labskin, UK using their proprietary reconstituted skin multi-well plate inserts comprised of adult human dermal fibroblasts embedded in fibrin with primary neonatal keratinocytes on the surface. Following tissue maturation, inserts were inoculated with 1x10^6^ CFU/cm^2^ of *C. acnes* and incubated at 37˚C in 5% (volume/volume) CO_2_ at >95% relative humidity for 1 hour to dry. 10 μL of BX001 gel or vehicle gel were applied to 3 inserts each at t=0 and to another 3 each at t=0 and again at t=24 hours. Additionally, 6 inserts were left untreated as controls of *C. acnes* growth. At 24- and 48-hours post-test item administration, 3 inserts of each group were assessed for bacterial burden. 5 mm biopsy samples were aseptically removed, and viable microbial numbers were assayed by enumeration of plaque formation on appropriate culture media at different dilutions of extracted bacteria. The level of detection of this assay was 10^2^ CFU/cm^2^. Statistical analyses on the results were performed using JMP® Pro Statistical Discovery software, version 15.2.1 from SAS® Institute Inc., Cary NC.

**Appendix S3. Safety evaluation methods.**

**Composite database and methods used in safety assessment of individual BX001 phages for undesired genes by in silico methods**

Coding sequences of phages in BX001 were screened against the following databases using BLAST^30^ with a cutoff of 30% global identity:

- **Toxin protein collection** – A collection of 7060 proteins collected from public protein databases, mainly NCBI (<https://www.ncbi.nlm.nih.gov/>) and the Animal toxin annotation project (<http://www.uniprot.org/program/Toxins>), according to a list of specific toxins in title 40 of the USA code of federal regulations endorsed by the USA Environmental Protection Agency^20^
- **Virulence genes**: Virulence genes were downloaded from The Lawrence Livermore National Laboratory Virulence Database (MvirDB; 94,068 entries). MvirDB is a database of microbial virulence factors - sequences representing known toxins, virulence factors, and antibiotic resistance genes. MvirDB integrates DNA and protein sequence information from [Tox-Prot](http://ca.expasy.org/sprot/tox-prot/) (SCORPION subset), the PRINTS database of virulence factors, VFDB, TVFac, Islander, ARGO, CONUS, KNOTTIN, a subset of VIDA and sequences derived by means of literature searches ^31^.
- **Antibiotic resistance genes**– Antibiotic resistance related genes were downloaded from CARD - The Comprehensive Antibiotic Resistance Database (2,158 entries). CARD is a bioinformatic database of resistance genes, their products and associated phenotypes ^32^.
- **Integrases**: Lysogeny, characterized by integration of the phage nucleic acids into the host bacterial genome, is mediated by phage-encoded integrases. All phage derived integrase proteins were collected from UniProt (<http://www.uniprot.org/>; 2,153 entries) using the keywords “integrase taxonomy:dsdna viruses”.
- **Generalized transduction by 16S ribosomal genes** – A database of 16S ribosomal RNA (Bacteria and Archaea) sequences was downloaded from NCBI (18,829 entries) to examine for the presence of bacterial derived sequences that could be indicative of the ability of phages to transfer bacterial sequences between bacteria, a phenomenon called generalized transduction that is not desired in phages intended for human application.

**Safety testing on reconstituted human tissues, EpiDerm^TM^ (MatTek) and EpiOcular™ (MatTek).**

Reconstructed human epidermal tissues (EpiDerm^TM^ , MatTek) consisting of normal human-derived epidermal keratinocytes cultured to form a multilayered highly differentiated model of the human epidermal layers and supplied as multiwell inserts were exposed to BX001 phages at high, medium and low concentrations for about 35 minutes and compared to tissues exposed to negative control (DPBS Rinse Solution, #TC-PBS, MatTek Corporation), positive control (5% SDS Solution, #TC-SDS-1, MatTek Corporation) and vehicle Sodium Magnesium (SM) buffer control with respect to relative viability. The amounts of BX001 Test Item were selected based on the intended maximal human exposure (high dose of BX001 over 200 cm^2^ of skin face surface) and calculated for the proportional dose in the EpiDerm™ tissues surface (0.63 cm^2^). The doses assayed, which were tested in triplicate, represent: 1X, 10X and 100X the maximal intended amounts for human exposure. Following dosing and incubation in Assay Medium, tissues were monitored for viability to determine possible irritation potential. Cell viability was measured by dehydrogenase conversion of MTT [(3-4,5-dimethyl thiazole 2-yl) 2,5-diphenyltetrazoliumbromide] into a blue formazan salt that is quantitatively measured after extraction from tissues. The assay was performed per manufacturer’s instructions and with reagents provided by the manufacturer.

Reconstructed human cornea-like epithelium tissues (EpiOcular™ MatTek) consisting of non-keratinized epithelium prepared from normal human keratinocytes which model corneal epithelium with progressively stratified, but not cornified, cells supplied as multiwell inserts were exposed in triplicate to BX001 phages at high (x100 Dose) and medium (x10 Dose) concentrations and compared to negative control (tissue culture grade water, #03-055-1A, Biological Industries), positive control (Methyl acetate, #TC-MA, MatTek Corporation) and vehicle control (Sodium Magnesium Buffer with respect to relative viability (%) as above.

**Assessment of phage permeation using an *ex vivo* human skin model**

To evaluate the potential of BX001 bacteriophages applied at the maximum intended concentration to permeate through to the dermis layer of the skin, a human epidermal sheet consisting of *stratum corneum* and epidermis was used for this study that was carried out in accordance to the principles of GLP as defined in the UK SI 1999 SI No 3106 (as amended by SI 2004 No 994) and the OECD principles of GLP (ENV/MC/CHEM. (98) 17).

Epidermal sheets were washed and positioned in sterilized vertical diffusion cells between a donor compartment and a receptor compartment with the stratum corneum facing the donor compartment. Following testing for integrity of the epidermal sheet by resistance measurement compared to a perturbed control, BX001 was introduced into 6 cells while two served as controls. After 1 hour no phage were detected in any of the receptor solutions. After 24 hours phages were detected at very low levels in the receptor compartment of 3 out of 6 of the vertical diffusion cells into which BX001 had been introduced (at 0.0069%, 0.0004% and 0.0163% of the applied dose as measured by 6 aliquots from each cell, although not all aliquots had phage).

**Appendix S4.** **Study design and methods of phase 1 cosmetic clinical trial of BX001 in mild-to-moderate acne.**

Study BMX-01-003 was a first-in-human, randomized, double-blind, vehicle-controlled, 3-arm parallel-group cosmetic study evaluating the safety, tolerability and effect of a topical cosmetic gel with BX001 cocktail of 3 phages targeted against *Cutibacterium acnes.* The study was conducted between October 2019 and January 2020 in a single center in Romania. The study protocol and informed consent form were reviewed by the Institutional Review Board of the site and all subjects provided written informed consent.

Key inclusion criteria included non-pregnant, non-lactating female subjects aged 18-40 at Screening, a clinical diagnosis of mild to moderate acne vulgaris with facial involvement with IGA score of 2-3, 5-40 inflammatory lesions on face (papules and/ or pustules) and no more than one active small nodular lesion, 25-50 non-inflammatory lesions on the face (open and/or closed comedones), sebum level > 110 µg/cm^2^, Fitzpatrick skin Phototype II-IV , willing to refrain from use of all other topical or systemic products for acne or any medicated topical preparations, during the study.

Key exclusion criteria included acne conglobata, acne fulminans, secondary acne (chloracne, drug-induced acne), or severe acne requiring systemic treatment, more than one active nodule on the face, any skin condition that would interfere with the diagnosis or assessment of acne vulgaris (including but not limited to rosacea, atopic dermatitis, psoriasis, squamous cell carcinoma, contact eczema acneiform eruptions caused by treatments, steroid acne, steroid folliculitis or bacterial folliculitis, on the face). Subjects were excluded if they used prior or concomitant treatment with systemic steroids, systemic antibiotics, systemic treatment for acne vulgaris, systemic anti-inflammatory agents within 4 weeks prior to Baseline or during the study ; topical steroids, topical antibiotics, topical treatment for acne vulgaris, topical anti-inflammatory agents or any hormonal topical treatment within 2 weeks prior to Baseline or during the study ; or isotretinoin or isotretinoin derivatives within 6 months prior to Screening.

Female subjects were randomized, in a 1 :1 :1 randomization ratio to daily administration of BX001 low dose, or BX001 high dose, or vehicle for 4 weeks, with a 1 week follow-up period. Subjects had 5 study visits: at Screening (Day –4 ± 2), Baseline (Day 0), 2 weeks (Day 14 ± 2), 4 weeks (Day 28 ± 3) and 5 weeks (Day 35 ± 2), with assessment of inflammatory and non-inflammatory lesions; quantitative polymerase reaction (qPCR) were collected using skin swabs. Each treatment was self-administered daily, at least 1 hour before bedtime, for 4 weeks, except for the Baseline visit when the application was carried out at the center in the presence of the study coordinator. Laboratory evaluations were completed at Screening (Day –4) and end of treatment (Day 28) and included hematology and serum chemistry tests. Urine pregnancy testing was completed on all subjects at Screening and final visit (Day 35). Safety was further assessed by physical examination of the face at all visits by dermatologist, vital signs (heart rate, systolic/diastolic blood pressure, temperature), adverse events and tolerability (erythema, edema, dryness or scaling assessed by the Investigator, and itching or burning / stinging assessed by the subject, on a 4-point scale with 0-none to 3-severe).

The primary endpoint was to evaluate the safety and tolerability of BX001. Exploratory endpoints included the effect of BX001 evaluated by the change of *C. acnes* on facial skin by quantitation of bacteria with specific *Cutibacterium* qPCR, changes in relative abundance of skin bacterial microbiome by V1-V3 16S rRNA DNA sequencing, change in total lesion count (non-inflammatory and inflammatory) and Investigator’s Global Assessment (IGA) score (from 0 to 4, with 0 = clear skin to 4 = severe acne), and subject satisfaction questionnaire.

Skin swabs from the cheeks were processed for bacterial DNA extraction and analysis using specific qPCR for *Cutibacterium* spp to evaluate the absolute amount of this bacteria and change from baseline and relative to vehicle after BX001 application. In addition, changes in microbial relative abundance of different species was evaluated by 16S rDNA V1-V3 sequencing. Only DNA of bacterial origin was analyzed.

In post-hoc analyses to assess the development of phage-resistant *C. acnes* during therapy with BX001, a plaque assay was performed on 211 *C. acnes* isolates obtained from swabs of subjects treated with low dose BX001, high dose BX001 or vehicle. Strains were considered sensitive to phage if they showed evidence of plaque-forming units (PFU).

Descriptive statistics are provided by group and overall, as relevant. Numerical variables were tabulated using mean, standard deviation, minimum, median, maximum and number of observations. Only observed data were used; missing data were not imputed. Categorical variables were tabulated using number of observations and percentages. Change from baseline (raw and percent) was analyzed using a mixed model repeated measures (MMRM) analysis of adjusted means, with Baseline qPCR as a covariate, Treatment group, Visit, and the interaction between the two as fixed effect factors. The model included random subject effect. All pairwise comparisons were carried out based on this model:

- BX001 low dose vs. placebo (vehicle)
- BX001 high dose vs. placebo (vehicle)

**Molecular methods for quantitation of *C.* *acnes* and determination of Shannon index of diversity**

Quantitation of *C. acnes* was carried out using a specific qPCR developed for *Cutibacteria* spp. DNA was extracted from swabs using the DNeasy PowerSoil kit (Qiagen). The swabs were added to PowerBead tubes containing 60 ul of Solution C1, incubated at 70^o^C for 10 min and vortexed at maximum speed for 10 min using a Vortex Adapter tube holder. Lysis and extraction steps were carried out manually, while loading on the column, washes and elution were done with the Qiacube HT according to the program recommended by the manufacturer. DNA was eluted with 90 ul Tris 10 mM.

Quantitative-PCR was carried out using specific primers and TaqMan MGB probe targeting a region common to all *Cutibacterium* species. Amplification and detection were performed with the LightCycler 480 (Roche Diagnostics) for 45 cycles. Every sample was run in triplicate. The LightCycler 480 v1.5 program (Roche Diagnostics) was used to calculate the quantity of *Cutibacterium* spp. (copies/sample) by using the second derivative method and a standard curve of Cq versus logarithm of the quantity. The standard curve was established using 11 known amounts of a 217 pb gBlock synthetic DNA from IDT Technologies (10 to 107 copies/reaction).

The analysis to examine changes in relative abundance of skin bacterial microbiome components used V1-V3 16S rRNA DNA sequencing. For the 16S V1-V3, tubes open to the environment were placed at the testing site to evaluate potential ambient contamination. Negligible amounts of bacterial reads were recovered from the open control tubes, with a different bacterial composition to the one obtained from the active and placebo groups, suggesting that there was no contamination of the study samples (data not shown).

Mock community analysis was conducted at the time of assay set up (data not shown). For determination of facial microbial composition, the V1-V3 variable region of the 16S rRNA was sequenced on an Illumina MiSeq PE250 platform using universal 16S primers.

Paired reads were trimmed, underwent quality checking and merged, then OTU (Operational Taxonomic Units) were picked and taxonomic assignation performed with the pick open reference otus.py script from Qiime1 v1.9.1^33^. The top 20 most abundant OTU at the genus and species levels for all samples were identified and the others were pooled. Diversity metrics and abundances were calculated using scripts from Qiime1 v1.9.1. The alpha diversity was computed with the alpha_diversity.py tool. Group comparisons were computed with the group_significance.py script.

The index selected to summarize the diversity of the bacterial population was the Shannon-Weiner diversity index because it is the most commonly used and it accounts for both the richness and the evenness of the population. Richness refers to the number of different bacteria in the samples. Evenness refers to the relative abundance between bacteria.

**Appendix S5. Additional results from phase 1 cosmetic clinical trial of BX001 in mild-to-moderate acne.**

Physical examination (limited to facial skin) was similar across treatment groups; there were no clinically significant abnormalities between baseline and subsequent visits. Analysis of vital signs did not reveal important differences between the treatment groups over time. There were no clinically significant abnormalities in hematology or chemistry test results post-screening. There was no significant difference in improvement in IGA score at any visit in any treatment group; all subjects were assessed as grade 2 or 3 IGA throughout the study period. The microbiome diversity as assessed by the Shannon index did not show a statistically significant change in alpha diversity in either BX001 group compared to vehicle at any timepoint.

A 9-item questionnaire to assess subject satisfaction with the product was administered at the End of Treatment Visit (Day 28). A greater proportion of subjects in both BX001 groups agreed that there was a significant clearing of skin (96% and 90.5%, respectively) compared to vehicle (79.2%). Also, more subjects in both BX001 groups agreed that the product improved skin texture (96% and 90.5%, respectively) compared to vehicle (83.3%) and more subjects in both BX001 groups agreed that the product absorbed quickly (100% and 95.2%, respectively), compared to vehicle (87.5%).

**Supplementary Figures**

**Figure S1**: ***C. acnes* counts in biofilm after treatment with phages or erythromycin.** Biofilm was treated with Cocktail 1 (Phages A and C plus an additional phage), Cocktail 2 (Phages A and B plus an additional phage), 4 phage Cocktail (Phages A, B and C plus an additional phage), 2µl of erythromycin (final concentration of 100 µg/mL) or no phage. Bacterial counts in the biofilm were assessed at 24 and 48 hours.

Biofilms, which constitute a common growth pattern of *C. acnes* and other bacteria, are complex structures consisting of bacterial colonies adhering to surfaces that secrete a mucilaginous protective coating in which they are encased. As biofilms are commonly considered to be resistant to antibiotics, the sensitivity of *C. acnes* growing in biofilm to phages was evaluated and compared to the activity of antibiotics. After *C. acnes* biofilm formation and 24 hours of phage treatment, a 4 log (10^4^) decrease in *C. acnes* bacterial counts is observed in all phage-treated arms relative to the untreated control. An additional log decrease was observed after an additional 24 hours of treatment. In contrast, the effect of erythromycin on bacteria growing in biofilm was observed only after 48 hours of treatment as an ~1 log reduction from baseline. These results demonstrate the efficacy of phages against target *C. acnes* bacteria even when these are growing within a biofilm. The different phage cocktails had similar strong activity against biofilm, suggesting that a robust biofilm penetrating ability is common across *C. acnes* phages. By comparison, the antibiotic erythromycin, to which this bacterial strain is sensitive, was significantly less effective in lysing *C. acnes* bacteria growing as biofilm and achieves only about a one log reduction in viable bacteria after 48 hours of exposure. Data represent mean ± SEM.

(a)
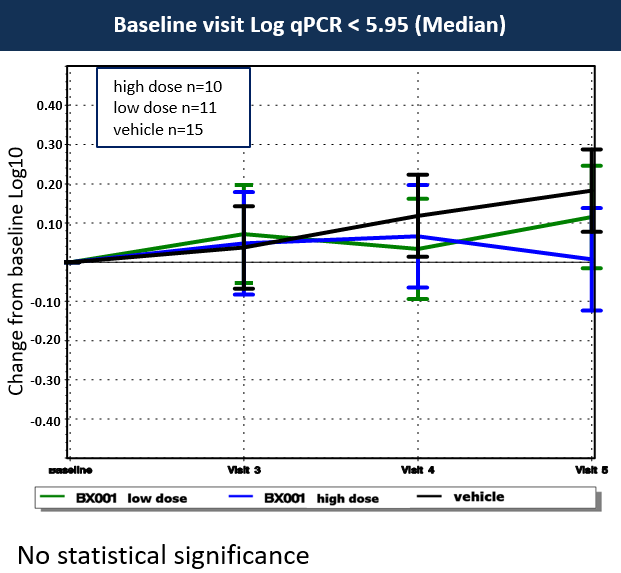
 (b)
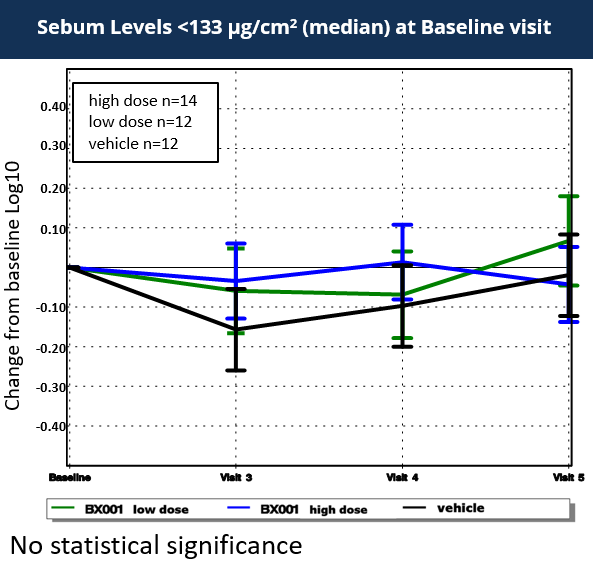


(c)
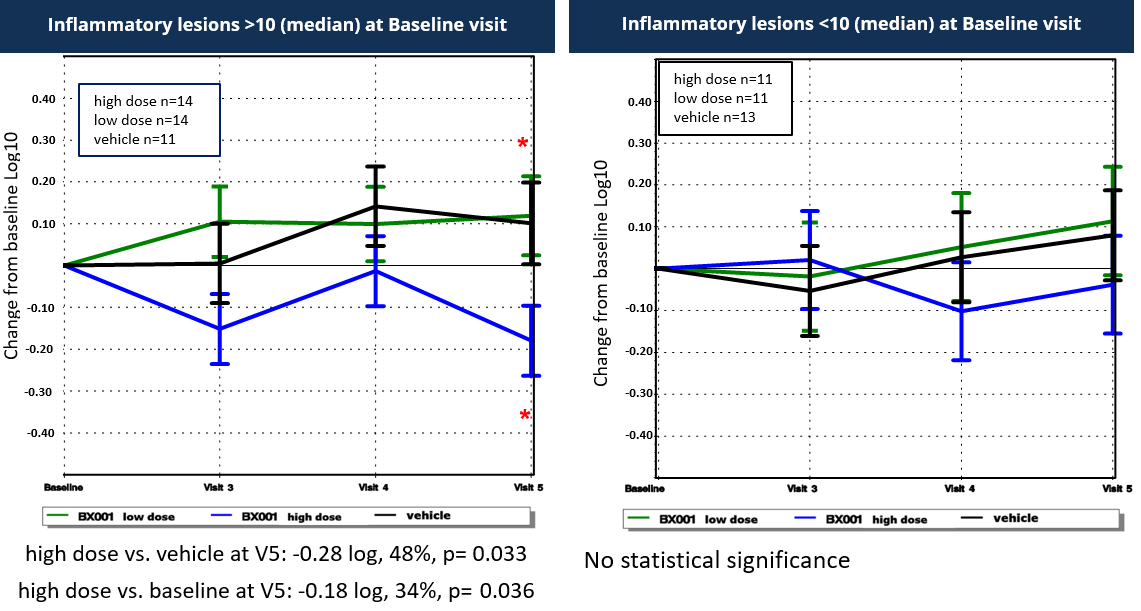


(d)
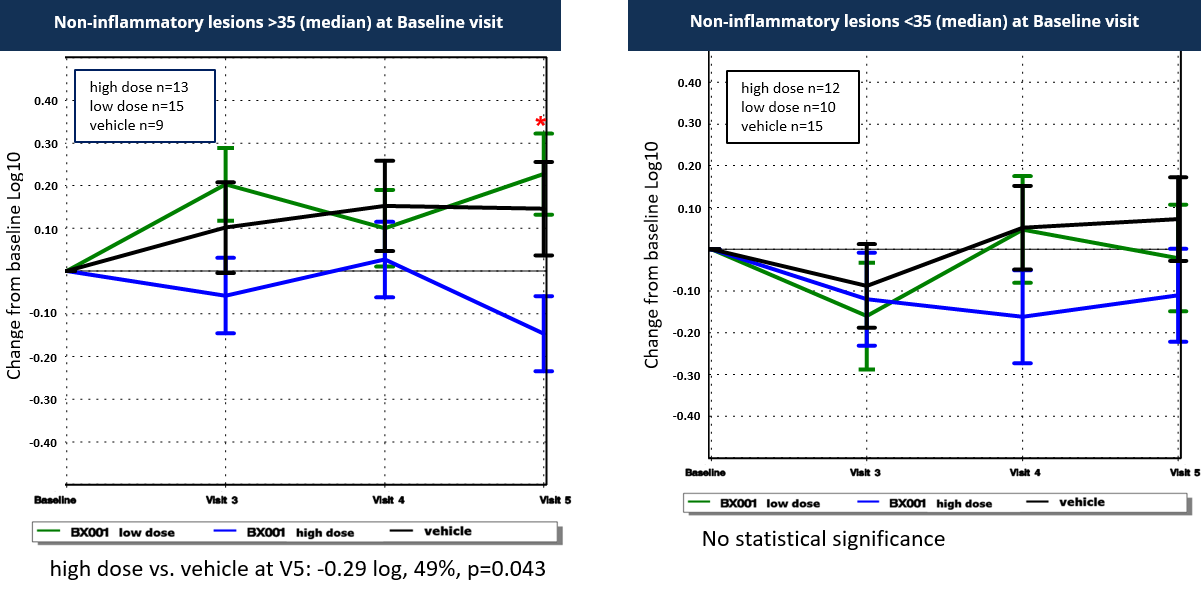


**Figure S2. Subgroup analyses of *Cutibacterium* qPCR response in phase 1 clinical trial (Full Analysis Set)**: (a) by baseline *Cutibacterium* bacterial burden below median (<5.95 log_10_); (b) by baseline sebum levels below median (<133 µg/cm^2^); (c) by baseline inflammatory lesions above median (>10) and below median (<10); (d) by baseline non-inflammatory lesions above median (>35) and below median (<35). Asterisk indicates a statistically significant difference between BX001 high dose group and vehicle (upper asterisk) or within BX001 high dose group compared to baseline value (lower asterisk). Data represent mean ± SEM.

**Supplementary Tables**

**Table S1.** **Characterization of *C. acnes* isolates obtained from acne vulgaris and healthy individuals showing acne severity by IGA assessment (ranging from 0: clear to 4: severe), bacterial ribotype, antibiotic sensitivity and sensitivity to BX001.** Both antibiotic resistant strains and strains representing ribotypes considered to be more frequently associated with acne were targeted by BX001. The BX001 susceptible strains included strains resistant to one of four tested antibiotics (Abx) frequently used in the treatment of acne (C=Clindamycin, T=Tetracycline, E=Erythromycin, M=Minocycline) and strains of different ribotypes confirmed by sequencing, including ribotypes 1, 2 and 3 which are equally found on acne and healthy individuals and representatives of ribotypes 4, 5, 8 and 9 (shown in cells shaded in blue) that are more highly associated with acne^3^.

Sample ID refers to the individual from whom bacteria were isolated. Boxes shaded in red indicate resistance to antibiotics or to phage. Clear boxes indicate sensitivity to these agents. Of 119 isolates tested, 24 (20%) displayed resistance to one or more antibiotics and only 5 (4%) displayed resistance to BX001. Blue boxes indicate ribotypes suggested to be disease associated; one of the blue boxes shows an isolate of ribotype 4 that is resistant to tetracycline but sensitive to BX001.

These findings validate the phage selection for inclusion in a highly efficacious phage cocktail.

| **Sample  ID** | **Acne severity  (IGA assessment)** | **Ribotype** | **Resistance** | |
| --- | --- | --- | --- | --- |
|  |  |  | **Abx** | **Phage** |
| 1 | Not available | RT3 | C |  |
|  |  | RT1 | C |  |
|  |  | RT3 | M |  |
|  |  | RT1 | M |  |
|  |  | RT3 | M |  |
| 2 | 2 | RT2 |  |  |
|  |  | RT2 |  |  |
|  |  | RT2 |  |  |
| 3 | 2 | RT1 |  |  |
|  |  | RT1 |  |  |
|  |  | RT1 |  |  |
| 4 | 2 | RT8 |  |  |
|  |  | RT8 | C and M |  |
|  |  | RT8 |  |  |
| 5 | 2 | RT1 |  |  |
| 6 | 2 | RT1 |  |  |
|  |  | RT1 |  |  |
| 7 | 1 | RT1 |  |  |
|  |  | RT2 |  |  |
|  |  | RT2 | E |  |
|  |  | RT2 | E |  |
|  |  | RT2 | E |  |
| 8 | 2 | RT1 |  |  |
|  |  | RT1 |  |  |
| 9 | 2 | RT5 |  |  |
| 10 | 0 | RT1 |  |  |
|  |  | RT2 |  |  |
| 11 | 2 | RT2 |  | BX001 |
|  |  | RT1 |  |  |
| 12 | 2 | RT1 |  |  |
|  |  | RT1 |  |  |
| 13 | 4 | RT3 |  |  |
|  |  | RT4 | T |  |
| 14 | 4 | RT1 |  |  |
|  |  | RT3 |  |  |
| 15 | 4 | RT1 | E | BX001 |
|  |  | RT2 |  |  |
|  |  | RT2 |  |  |
|  |  | RT1 |  | BX001 |
| 16 | 4 | RT2 |  |  |
| 17 | 0 | RT6 |  |  |
|  |  | RT2 |  |  |
| 18 | 0 | T838C, C1229T |  |  |
|  |  | RT2 |  |  |
| 19 | 0 | RT1 |  |  |
|  |  | RT1 |  |  |
| 20 | 0 | A434G, T838C |  |  |
|  |  | RT6 |  |  |
| 21 | 0 | RT3 |  |  |
|  |  | RT2 |  |  |
|  |  | RT2 |  |  |
| 22 | 3 | RT3 |  |  |
|  |  | RT3 | E |  |
|  |  | RT3 | C |  |
| 23 | 0 | RT1 |  |  |
|  |  | RT2 |  |  |
|  |  | RT1 | T |  |
|  |  | RT1 | C |  |
|  |  | RT1 | C |  |
|  |  | RT1 | E |  |
| 24 | 0 | RT1 |  |  |
| 25 | 1 | RT3 |  |  |
|  |  | RT2 |  |  |
|  |  | RT3 |  |  |
| 26 | 0 | RT2 |  |  |
| 27 | 2 | RT2 |  |  |
|  |  | RT3 |  |  |
|  |  | RT1 |  |  |
| 28 | 2 | RT9 |  |  |
|  |  | RT2 |  |  |
|  |  | RT3 |  |  |
| 29 | 2 | RT2 |  |  |
| 30 | 2 | RT2 |  |  |
|  |  | RT2 |  |  |
|  |  | RT3 |  |  |
| 31 | 2 | RT2 |  |  |
|  |  | RT1 |  |  |
|  |  | RT3 |  |  |
| 32 | 0 | RT2 |  | BX001 |
| 33 | 2 | RT2 |  |  |
|  |  | RT1 |  |  |
|  |  | RT2 |  |  |
|  |  | RT2 |  | BX001 |
| 34 | 2 | RT1 |  |  |
| 35 | 4 | RT2 |  |  |
|  |  | RT2 |  |  |
| 36 | 2 | RT2 |  |  |
|  |  | RT2 |  |  |
| 37 | 1 | RT2 |  |  |
|  |  | RT1 |  |  |
| 38 | 2 | RT1 | C |  |
|  |  | RT1 | C |  |
|  |  | RT1 | E |  |
| 39 | 1 | RT3 |  |  |
| 40 | 2 | RT1 |  |  |
|  |  | RT3 | C |  |
|  |  | RT3 | E |  |
| 41 | 2 | RT2 |  |  |
|  |  | RT3 |  |  |
|  |  | RT1 |  |  |
| 42 | 1 | RT3 |  |  |
|  |  | RT2 |  |  |
| 43 | 2 | RT2 |  |  |
| 44 | 1 | RT1 |  |  |
|  |  | RT1 |  |  |
| 45 | 0 | RT2 |  |  |
|  |  | RT3 |  |  |
|  |  | RT3 |  |  |
| 46 | 0 | RT2 |  |  |
|  |  | RT3 |  |  |
|  |  | RT3 | E |  |
|  |  | RT3 | C |  |
| 47 | 1 | RT1 |  |  |
| 48 | 0 | RT3 |  |  |
|  |  | RT3 |  |  |
| 49 | 0 | RT2 |  |  |
|  |  | RT2 |  |  |
| 50 | 0 | RT3 |  |  |
|  |  | RT2 |  |  |

**Table S2. Sensitivity of common skin bacterial species to each phage in BX001 as part of safety evaluation**. Phages generally exhibit species specificity. We evaluated the infection capacity of the BX001 phages on commercially sourced representative strains of the most common skin bacteria in order to assess the specificity of BX001 for *C. acnes*. Only *C. acnes* strains were found to be susceptible to the phages. Thus, unlike antibiotics, application of BX001 will not reduce the commensal population of beneficial bacterial species found on skin, such as *Staphylococcus epidermidis*.

| **Bacteria** | | | **Phages** | | |
| --- | --- | --- | --- | --- | --- |
| **Name** | **Origin** | **Code** | **A** | **B** | **C** |
| *Cutibacterium acnes* | CCUG 6369 | PA1 | + | + | + |
| *Cutibacterium acnes* | CCUG 38584 | PA7 | + | + | + |
| *Cutibacterium acnes* | DSMZ 16379 | PA13 | + | + | + |
| *Cutibacterium granulosum* | CCUG 32987 | PGr1 | - | - | - |
| *Cutibacterium avidum* | CCUG 36754 | PAv1 | - | - | - |
| *Staphylococcus aureus* | CCUG 41582 | SAu1 | - | - | - |
| *Staphylococcus epidermidis* | CCUG 21989 | SEp1 | - | - | - |

**Table S3. Baseline characteristics in phase 1 clinical trial (FAS Population).**

| **Baseline Characteristic** | **BX001 low dose**  **(N=25)** | **BX001 high dose**  **(N=25)** | **Placebo (Vehicle)**  **(N=25)** |
| --- | --- | --- | --- |
| Age (years, SD) | 24.6 (6.1) | 25.7 (7.3) | 23.4 (4.4) |
| Fitzpatrick phototype (percent of 2/3) | 52/48 | 32/68 | 56/44 |
| IGA Score (percent of 2/3) | 80/20 | 96/4 | 87.5/12.5 |
| Inflammatory lesions (number, SD) | 12.0 (7.2) | 11.6 (5.9) | 10.3 (4.8) |
| Non-inflammatory lesions (number, SD) | 36.4 (7.7) | 36.1 (6.9) | 35.2 (8.6) |
| log_10_ qPCR copies of *Cutibacteria*/sample (mean, SD) | 6.08 (0.4) | 6.01 (0.45) | 5.94 (0.45) |

FAS : Full Analysis Set

**Table S4. Adverse events by treatment group in phase 1 clinical trial (Safety Population).**

Treatment-related adverse events included adverse events that were classified as either “Probably” or “Possibly” related by the Investigator. The most common treatment-related AE was skin exfoliation, occurring in 4 subjects in each BX001 treatment group: all cases were mild in severity, lasted 1 day or less, and resolved completely without requiring any additional treatment.

| **Adverse Events** | **BX001 low dose**  **(N=25)** | | **BX001 high dose**  **(N=25)** | | **Placebo (Vehicle)**  **(N=24)** | |
| --- | --- | --- | --- | --- | --- | --- |
|  | **N** | **%** | **N** | **%** | **N** | **%** |
| Number of AEs | 25 |  | 20 |  | 27 |  |
| Number of subjects with : | | | | | | |
| All causality AE | 13 | 52.0 | 11 | 44.0 | 10 | 41.7 |
| Treatment-related AE | 7 | 28.0 | 6 | 24.0 | 4 | 16.7 |
| Premature discontinuation from the study due to AE | 0 | 0.0 | 0 | 0.0 | 0 | 0.0 |
| Serious AEs | 0 | 0.0 | 0 | 0.0 | 0 | 0.0 |
| Deaths | 0 | 0.0 | 0 | 0.0 | 0 | 0.0 |

**Table S5. Tolerability at end of treatment in phase 1 clinical trial (PP Population) ***

|  | | **BX001 low dose**  **n (%)** | | **BX001 high dose**  **n (%)** | | **Placebo (Vehicle)**  **n (%)** | |
| --- | --- | --- | --- | --- | --- | --- | --- |
|  |  | **None** | **Mild** | **None** | **Mild** | **None** | **Mild** |
| Investigator Assessment | Erythema | 21 (100) | 0 | 24 (96) | 1 (4) | 24 (100) | 0 |
|  | Edema | 21 (100) | 0 | 25 (100) | 0 | 24 (100) | 0 |
|  | Dryness | 21 (100) | 0 | 25 (100) | 0 | 24 (100) | 0 |
|  | Scaling | 21 (100) | 0 | 24 (96) | 1 (4) | 24 (100) | 0 |
| Subject Assessment | Itching | 21 (100) | 0 | 25 (100) | 0 | 24 (100) | 0 |
|  | Burning | 21 (100) | 0 | 25 (100) | 0 | 24 (100) | 0 |

Figure legend PP: Per Protocol

*The tolerability analysis was evaluated by the Investigator using 4 parameters and by the subjects using 2 parameters.
